# Preoperative Botulinum Toxin A and Its Impact on Pulmonary Function in Giant Abdominal Wall Hernias: A Prospective Spirometry-Based Analysis

**DOI:** 10.3389/jaws.2025.14753

**Published:** 2025-06-24

**Authors:** Maciej Śmietański, Mateusz Zamkowski, Irmina Anna Śmietańska, Michał Putko, Orest Lerchuk, Andriy Porytsky, Zhanna Ushnevych, Volodymyr Khomyak

**Affiliations:** ^1^ Swissmed Hospital, Gdańsk, Poland; ^2^ II Department of Radiology, Medical University of Gdansk, Gdańsk, Pomeranian, Poland; ^3^ Lviv Regional Clinical Hospital, Lviv, Ukraine; ^4^ Danylo Halytsky Lviv National Medical University, Lviv, Ukraine

**Keywords:** botulinum toxin A, abdominal wall hernias, pulmonary function, spirometry, abdominal wall repair

## Abstract

**Background:**

Botulinum toxin type A has become an increasingly used tool in the preoperative management of giant abdominal wall hernias. Its primary objective is to “downstage” the hernia by inducing temporary paralysis of the lateral abdominal wall muscles, thereby increasing their compliance and enabling safer fascial closure. While the muscular and anatomical benefits of this approach are well documented, the potential effects on pulmonary function remain poorly studied, despite the involvement of the targeted muscles in the process of breathing.

**Objective:**

This study aimed to evaluate the impact of botulinum toxin type A on respiratory system function, using spirometry to assess whether any observed changes reflect true improvement, mechanical compensation, or potential impairment.

**Methods:**

This prospective, observational study included 37 patients with large abdominal wall hernias and a Loss of Domain component. All patients received 300 units of botulinum toxin type A injected bilaterally into the external, internal oblique, and transversus abdominis muscles under ultrasound guidance. Spirometry was performed before the injection and again on the day of surgery. Evaluated parameters included forced vital capacity, forced expiratory volume in one second, the ratio of forced expiratory volume to forced vital capacity, peak expiratory flow, maximum mid-expiratory flow, maximal expiratory flow at 75, 50, and 25 percent of forced vital capacity, forced inspiratory vital capacity. Results were analyzed using paired statistical tests with a significance threshold of p < 0.05.

**Results:**

No statistically significant changes were observed in forced vital capacity or forced expiratory volume in one second. However, statistically significant increases were recorded in maximum mid-expiratory flow and maximal expiratory flow at 50 percent of lung volume. Peak expiratory flow showed a trend toward improvement but did not reach statistical significance. These changes appear to reflect altered expiratory dynamics due to increased diaphragmatic excursion, rather than improved ventilation. Forced inspiratory vital capacity decreased slightly. Only two patients reported subjective changes in breathing.

**Conclusion:**

Botulinum toxin type A does not impair core lung volumes but induces mechanical changes that may affect airflow velocity. Standard spirometry may not fully reflect these dynamics, and further investigation is warranted to better understand respiratory outcomes in this patient group.

## Introduction

The primary goal of optimal midline hernia repair is to achieve complete defect closure, restore the linea alba, and approximate the rectus muscles. While this represents the ideal outcome, it is not always feasible, especially in cases of giant hernias with a Loss of Domain component. In such situations, alternative techniques must be employed to bring us closer to the desired goal, often requiring certain compromises [1].

Avoiding the need for “bridging” mesh repair relies on both preoperative and intraoperative strategies. Intraoperative approaches primarily include “component separation” techniques, but newer tools, such as the Fasciotens^®^ (GmbH^®^, Germany) device, are also gaining popularity [2, 3].

Preoperative preparation has increasingly incorporated botulinum toxin type A (BTA) injections into the lateral abdominal muscles. This method aims to induce temporary paralysis of these muscles, enhancing their compliance and facilitating the approximation of fascial edges for defect closure. [4–6]. One of the key advantages of BTA over component separation techniques is the temporary nature of its effects—once the BTA wears off, normal muscle function is expected to return. Unlike Anterior or Posterior Component Separation, BTA application does not require the permanent division of muscle attachments, thereby reducing intraoperative trauma and minimizing postoperative complications such as hematomas, seromas, and necrosis of skin flaps [7–9]. Additionally, compared to progressive preoperative pneumoperitoneum, BTA injections have fewer reported adverse effects in the literature [10–12].

However, this does not imply that BTA use is entirely risk-free. Rather, there is a lack of comprehensive scientific data on its potential negative effects, particularly on the pulmonary system. Although BTA injections are generally regarded as a safe and simple procedure, occasional reports of severe adverse effects have emerged [13, 14].

Most importantly, in the light of above cited paper [13], it remains uncertain whether BTA is equally beneficial for all patients or if there exists a subgroup in which negative outcomes outweigh the positive effects. This question is particularly relevant in the context of the respiratory system, as the lateral abdominal muscles play an active role in breathing.

To address this concern, we designed an observational, prospective, two-center clinical study, assessing respiratory function via spirometry in patients undergoing abdominal wall reconstruction. The aim of this study was to evaluate the impact of botulinum toxin type A on respiratory system function using spirometry.

## Materials and Methods

The study was conducted in two centers: Swissmed Hospital in Gdańsk and the Surgical Clinic in Lviv. The enrolled patients had giant abdominal hernias with a Loss of Domain component (inclusion and exclusion criteria are provided in Table 1).

**TABLE 1:** Inclusion and exclusion criteria.

Inclusion criteria	Exclusion criteria
Large abdominal hernia (at least W3 in the EHS classification), requiring additional preoperative techniques to prevent abdominal compartment syndrome (ACS) in the postoperative period	Refusal to participate in the study
Age >18 years	
Written consent to participate in the study	
Health status allowing the safe conduct of surgery	

All patients underwent spirometry testing on the day of BTA administration and again on the day of surgery. Preoperative BTA injections were administered 4–6 weeks before surgery. The study included 8 patients from Lviv and 29 from Gdańsk. In addition to standard demographic data, detailed information regarding comorbidities and hernia characteristics was collected. Demographic data were gathered prospectively at the time of patient enrolment. The most common comorbidities among the 37 enrolled patients included hypertension (n = 21), type II diabetes mellitus (n = 8), hypothyroidism (n = 4), atrial fibrillation (n = 2), chronic venous insufficiency (n = 2), gastroesophageal reflux disease (n = 2), asthma (n = 1), chronic obstructive pulmonary disease (n = 1), chronic pancreatitis (n = 1), and systemic lupus erythematosus (n = 1). Hernias were classified according to the European Hernia Society (EHS) criteria, with the most frequent types being M2–M4W3 (n = 10), M1–M4W3 (n = 9), and M2–M5W3 (n = 5). Additionally, three patients presented with parastomal hernias (two type III, one type IV), and one patient had a giant scrotal hernia with loss of domain. The mean transverse diameter of the hernia defects was 14.6 cm (SD ± 3.19), reflecting the complexity and magnitude of abdominal wall impairment in this cohort (Table 2). The procedure was performed in an outpatient setting under ultrasound guidance to ensure accurate needle placement. Each patient received 300 units of BTA (Dysport^®^, IPSEN, Boulogne-Billancourt, France), with 150 units injected per side. During preparation for BTA administration, the lower edge of the last rib and the upper border of the iliac crest were marked on the skin. A line was drawn along the anterior axillary line, connecting these two points, and three evenly spaced injection sites were identified. A total of 300 units of BTA were diluted in 150 mL of 0.9% saline. The solution was then divided into six portions of 25 mL each. Under ultrasound guidance, the needle was inserted into the transverse abdominal muscle, where 8 mL of solution was administered. The needle was then partially withdrawn, and another 8 mL was injected into the internal oblique muscle. Finally, after further withdrawal, the remaining portion of the dose was delivered into the external oblique muscle. This procedure was repeated at each designated injection site. The described method has been previously documented in the scientific literature [9].

**TABLE 2:** Demographic characteristics of the study Population general information.

Demographic Variable	Value
Age (years)	58.81 ± 12.82
BMI (kg/m^2^)	30.59 ± 4.14
Comorbidities	Hypertension – 21 Gastroesophageal reflux – 2 Diabetes Melitus type II – 8 Chronic Venous Insufficiency – 2 Hypothyroidism – 4 Systemic Lupus – 1 Asthma - 1 Chronic Pancreatitis – 1 Atrial Fibration – 2 COPD - 1
Hernia Types (EHS Midline Hernia Classification and EHS Parastomal Hernia Classification)	M1-M5W3 – 4 M1-M4W3 – 9 M1-M3W3 - 1 M2-M4W3 – 10 M2-M5W3 – 5 Parastomal Hernia type III – 2 Parastomal Hernia type IV - 1 L2-L3W3 (left side) – 2 L2W3 (right side) – 1 M2-M3W3 + L3W3 (left side) – 1 Giant scrotal hernia (type S3, LOD) right side - 1
Mean Defect Size (transverse diameter, SD)	14.6 (3.19)
Average interval between spirometry tests (weeks)	6.26 ± 2.38
Sex (female)	26
Gdansk Clinic	29/37

Abbreviations: COPD, Chronic obstructive pulmonary disease; LOD, loss of domain; EHS, European Hernia Society.

All patients underwent pulmonary function testing using spirometry on the day of botulinum toxin type A (BTA) injection and again on the day of planned hernia repair surgery. Spirometry was performed in accordance with American Thoracic Society (ATS) and European Respiratory Society (ERS) standards using a portable spirometer (BTL-08 Spiro Pro, BTL Industries Limited, Great Britain). Each test was performed in a seated position, with a nose clip, and following standard instructions for maximal inspiration and expiration. At least three acceptable maneuvers were required for each test, with the best result used for further analysis.

The spirometry parameters evaluated included:

Forced Vital Capacity (FVC) – the total volume of air that can be forcibly exhaled after full inspiration;
Forced Expiratory Volume in 1 Second (FEV1) – the volume of air expelled in the first second of the FVC maneuver;
FEV1/FVC ratio–expressed as a percentage, assessing airflow limitation;
Peak Expiratory Flow (PEF) – the maximum flow achieved during forced expiration;
Maximum Mid-Expiratory Flow (MMEF) – the average flow rate during the middle half of the FVC maneuver;
Maximal Expiratory Flow at 75%, 50%, and 25% of FVC (MEF75, MEF50, MEF25) – representing flow rates at different lung volumes;
Forced Inspiratory Vital Capacity (FIVC) – the total inspiratory capacity following a maximal expiration;
AEX (Area Under the Expiratory Flow–Volume Curve) – a spirometric parameter calculated as the total area beneath the expiratory flow–volume curve. It integrates both flow and volume to provide a composite measure of expiratory performance, reflecting the overall effort and efficiency of expiration.

Additionally, patients were asked a qualitative question regarding perceived changes in respiratory status after BTA injections: “Did you observe or feel any changes in your breathing function or physical exertion capacity?.”

The data were subjected to statistical analysis using paired t-tests to compare pre- and post-injection values. Statistical significance was set at p < 0.05. For each parameter, the following were reported: mean values, standard deviation, and full range (minimum–maximum). When applicable, confidence intervals (CI) and odds ratios (OR) were calculated. Statistical analysis was performed using STATISTICA (data analysis software system, StatSoft. Inc. 2014. version 12.0).

The study was registered on ClinicalTrials.gov under the number NCT06485440, and received ethical approval from the Bioethics Committee by Regional Medical Chamber in Gdańsk (KB - 42/23).

### Analysis of Spirometric Parameters in the Context of Respiratory Mechanics

In order to interpret the spirometric parameters described following the analysis of the recorded results, it is necessary to first explain the physiological mechanisms of inspiration and expiration [15].

#### The Mechanism of Inspiration Is Illustrated in Figure 1

Inspiration is an active process dependent on the simultaneous contraction of the diaphragm—which increases the thoracic volume by pulling the lungs downward—and the accessory muscles, namely the intercostal muscles and the sternocleidomastoid, which expand the thoracic cavity by lifting the ribs laterally and superiorly. The position of the diaphragm at the start of inspiration is high; during contraction, it flattens its dome-shaped curve, shortens, and descends. The rectus abdominis and lateral abdominal wall muscles relax, allowing the abdominal contents to be displaced further into the abdominal cavity.

**FIGURE 1:**
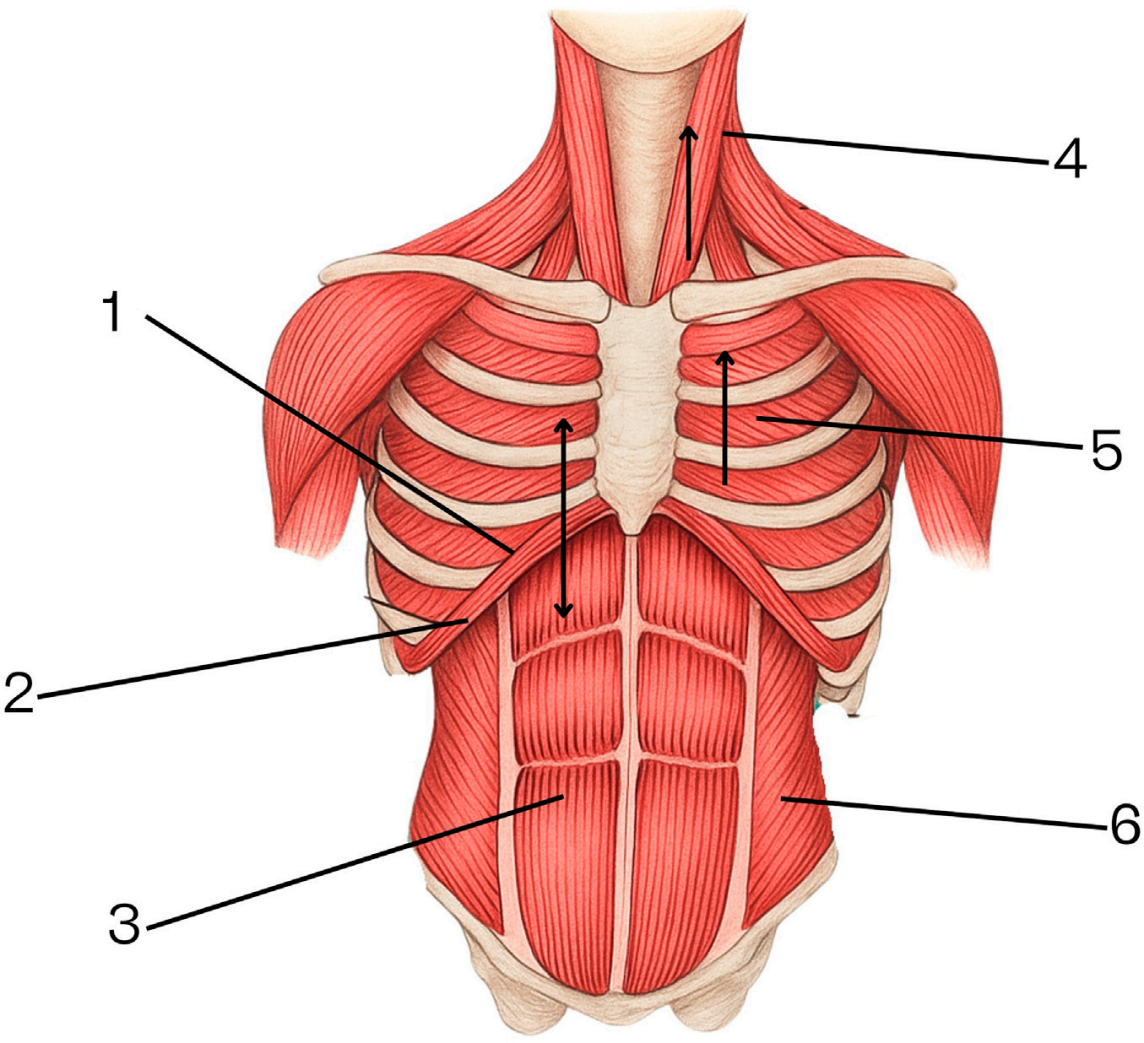
Mechanism of inspiration. 1. Position of the diaphragm at the beginning of inspiration. 2. Position of the diaphragm at the end of inspiration. 3. Rectus abdominis muscles. 4. Sternocleidomastoid muscle. 5. Intercostal muscles (anterior and posterior). 6. Lateral abdominal wall muscles (transversus abdominis, internal oblique, external oblique).

#### The Mechanism of Expiration Is Illustrated in Figure 2

Expiration is also an active process. It is important to note that the parameters assessed in spirometry do not reflect the mechanics of quiet breathing. During testing, the patient is asked to perform a maximal exhalation. The intercostal muscles and the sternocleidomastoid relax, allowing the thoracic cage to descend due to gravity, reversing the expansion that occurred during inspiration. The diaphragm also relaxes, becoming more compliant to external pressure—in this case, the increase in intra-abdominal pressure. This increase in intra-abdominal pressure is caused by the synchronous contraction of the rectus abdominis muscles (as the primary group) and the lateral abdominal muscles (as secondary contributors). This coordinated action pushes the diaphragm upward, reducing lung volume. The peak position of the diaphragm at the end of expiration is determined by the strength of this combined muscle contraction.

**FIGURE 2:**
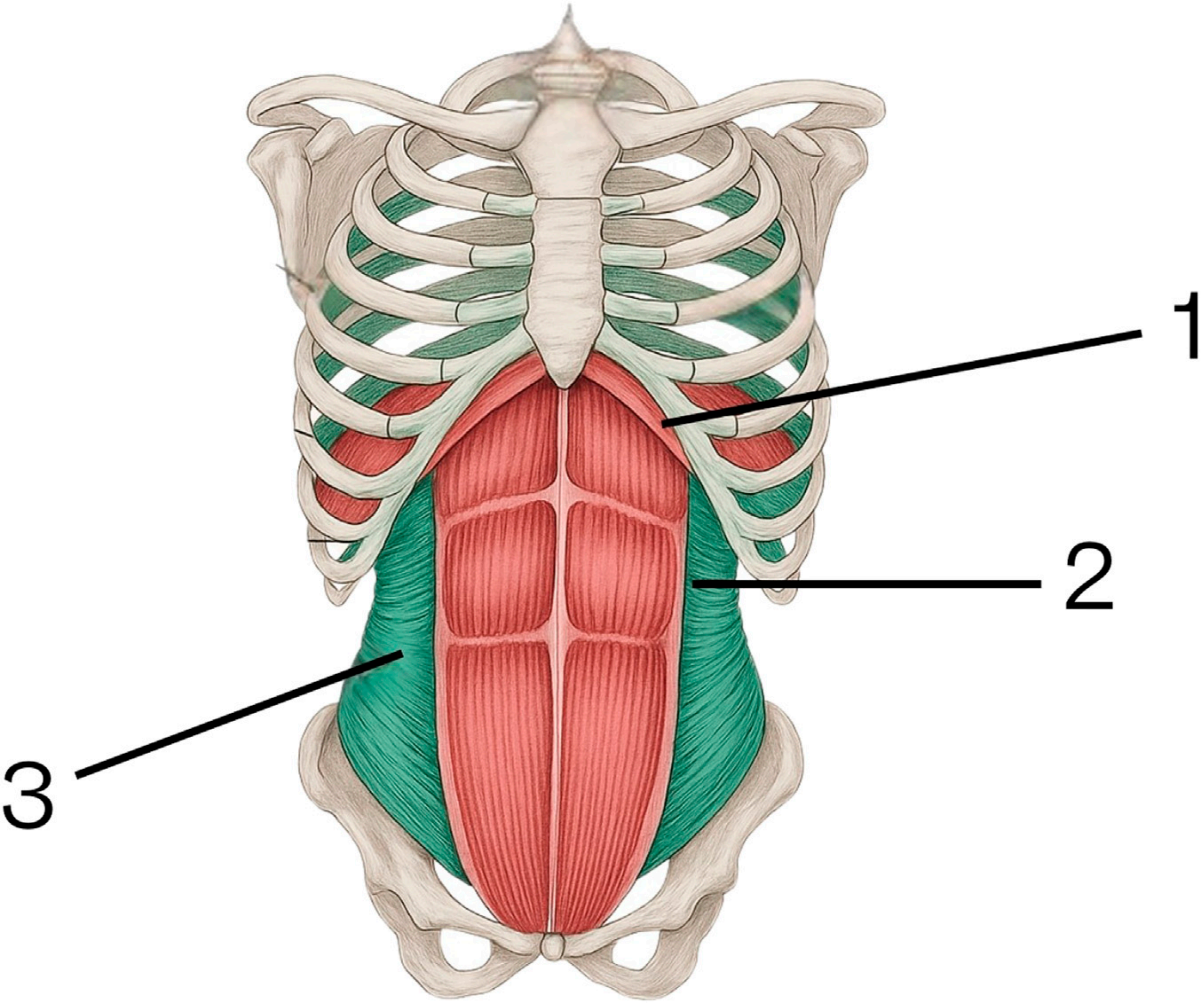
Mechanism of expiration. 1. Diaphragm. 2. Rectus abdominis muscles. 3. Lateral abdominal muscle group.

The potential impact of botulinum toxin administration—i.e., the temporary paralysis of the lateral abdominal muscle group—on the described mechanics of inspiration and expiration must be considered.

Based on the mechanisms outlined above, it can generally be stated that the administration of botulinum toxin does not affect the mechanics of inspiration. The key muscles responsible for this phase of breathing remain fully functional and are not subject to the effects of BTA.

In the expiratory phase, however, the following changes can be observed:

1. In the initial phase of expiration, the auxiliary activity of the lateral abdominal muscles is no longer present. Since these muscles serve only a supportive function, their inactivity does not significantly affect the FEV1 value. The observed increase of 1.95% was not statistically significant and may reflect natural variability or be caused by leak of lateral muscles activity as effect of BTA (rather than a physiologic effect of BTA).
2. The parameters PEF and MMEF show improvement (MMEF changes were statistically significant). This can be explained by the increased initial lung volume, which leads to a greater pressure gradient during the early and middle phases of expiration. This increased gradient originates from the greater expansion of the lungs at the beginning of inspiration (not expiration), which in turn results from passive stretching of the lateral abdominal muscles. This stretching enhances the downward pull on the diaphragm during the initial phase of inspiration (see point 3 below). As a result, a larger volume of air can be expelled more easily in the early and middle phases of expiration using only the rectus abdominis muscles. In the described spirometry data, PEF and MMEF increased by approximately 10.5%. This moderate increase reflects changes in airflow velocity, not rather than a true improvement in overall respiratory efficiency (corelated with no changes in FEV).
3. The paralysis of the lateral muscle group eliminates the third phase of expiration. The abdominal viscera have considerable mass, and without active contraction of the lateral abdominal muscles, the compressive action of the rectus abdominis alone is insufficient to elevate the abdominal contents. As a result, the diaphragm settles in a lower position at the end of expiration. Although the residual lung volume increases, the amplitude of lung volume variation is reduced. The observed increase of 1.95% - which was not statistically significant and may reflect normal variability - suggests that the absence of lateral abdominal muscle activity has minimal impact on FEV1.

**FIGURE 3:**
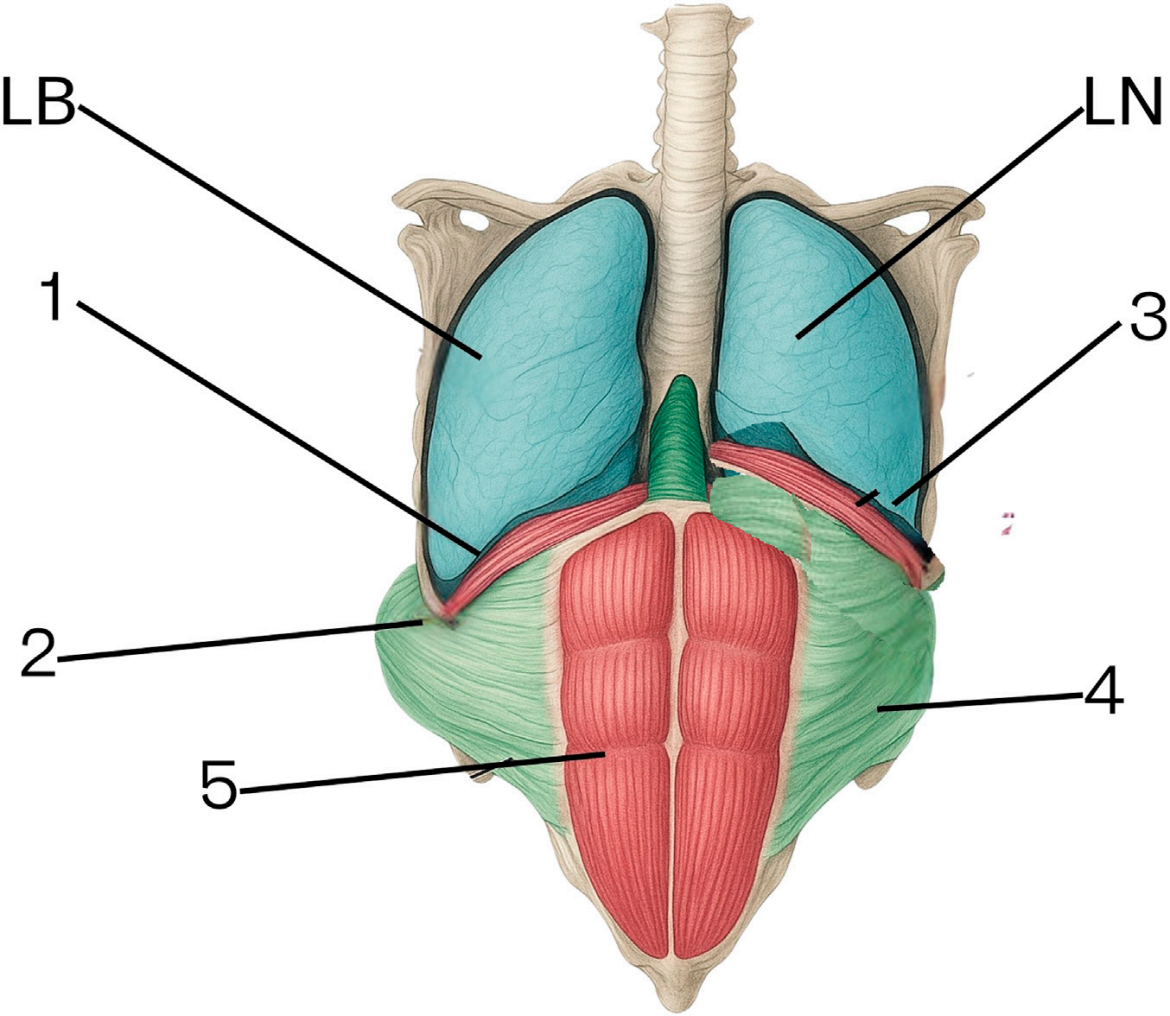
Diagram of Diaphragm Positioning and Abdominal Mechanics Post-BTA Injection. 1. Diaphragm position at the end of expiration following botulinum toxin administration. 2. Passive stretching of the lateral abdominal wall creating more space for the abdominal viscera. 3. Diaphragm position at the end of inspiration under natural (non-BTA) conditions. 4. Lateral abdominal muscle position at the end of inspiration under natural (non-BTA) conditions. 5. Rectus abdominis muscles (function preserved in both conditions). 6. LB – representation of lung volume at the end of expiration post-BTA. 7. LN – representation of lung volume at the end of expiration without BTA.

This process is illustrated in Figure 3.

## Results

The mean forced vital capacity (FVC) prior to injection was 5.51 L, with a range from 2.17 to 11.64 L. After BTA administration, the mean FVC was 5.54 L (range: 2.24–12.55), with no statistically significant difference (p = 0.7910). Similarly, the forced expiratory volume in one second (FEV1) increased slightly from a mean of 4.61 L (range: 1.74–9.56) to 4.70 L (range: 2.14–10.90), which was also not statistically significant (p = 0.2995). The FEV1/FVC ratio improved modestly, increasing from 81.56% (range: 58.29–100.0) to 84.44% (range: 65.66–100.0), but this change did not reach statistical significance (p = 0.0823).

Peak expiratory flow (PEF), which represents the maximal speed of exhalation, increased from a mean value of 8.82 L per second (range: 4.25–15.49) before BTA injection to 9.75 L per second (range: 3.55–16.85) post-injection. This difference approached, but did not reach, statistical significance (p = 0.0722).

Statistically significant changes were observed in parameters associated with airflow in the smaller airways. The maximum mid-expiratory flow (MMEF) increased from a mean value of 5.02 L per second (range: 1.76–10.28) to 5.55 L per second (range: 1.98–13.38), with a p-value of 0.0168. A similar significant improvement was noted for MEF50, which rose from 5.29 L per second (range: 2.06–11.27) to 5.94 L per second (range: 2.27–15.61), with a p-value of 0.0234. For MEF75 and MEF25, the observed increases were not statistically significant.

The AEX parameter, measured in a subset of patients, increased from 30.75 (range: 4.86–92.96) to 35.31 (range: 7.02–132.77), with a statistically significant difference (p = 0.0259). All data are presented in Table 3. Subjective assessment was also collected. Patients were asked whether they perceived any change in their breathing or physical exertion capacity between the two timepoints. The results of this self-assessment are presented as a supplementary in Table 3 and were not subjected to statistical testing in this study phase. No adverse respiratory events were reported during the period between BTA administration and surgery. Spirometric testing was completed without complications in all participants.

**TABLE 3:** Extended spirometry summary Table.

Parameter	Mean pre-botox (range)	Mean post-botox (range)	Mean difference	95% CI	p-value
FVC	5.51 (2.17–11.64)	5.54 (2.24–12.55)	0.03	−0.19 to 0.25	0.791
FEV1	4.61 (1.74–9.56)	4.70 (2.14–10.90)	0.09	−0.08 to 0.25	0.2995
FEV1/FVC	81.56 (58.29–100.0)	84.44 (65.66–100.0)	2.88	−0.39 to 6.16	0.0823
PEF	8.82 (4.25–15.49)	9.75 (3.55–16.85)	0.92	−0.09 to 1.94	0.0722
MMEF	5.02 (1.76–10.28)	5.55 (1.98–13.38)	0.53	0.10 to 0.96	0.0168
MEF75	5.97 (2.51–12.20)	6.13 (2.42–13.26)	0.16	−0.29 to 0.62	0.3535
MEF50	5.29 (2.06–11.27)	5.94 (2.27–15.61)	0.65	0.10 to 1.19	0.0234
MEF25	3.32 (1.23–8.92)	3.55 (0.87–10.05)	0.23	−0.20 to 0.66	0.2613
AEX	30.75 (4.86–92.96)	35.31 (7.02–132.77)	4.56	0.65 to 8.48	0.0259
FIVC	4.60 (1.77–8.59)	4.48 (0.65–8.39)	−0.12	−0.39 to 0.15	0.3677
**Self-reported respiratory changes after botulinum toxin injection**					
Question					Answer: yes
Did you observe or feel any changes in your breathing function or physical exertion capacity?”					2/37

In summary, BTA administration did not lead to a significant decline in primary lung function parameters such as FVC or FEV1. Improvements were observed in MEF50, MMEF, and AEX values, with statistically significant changes noted in some cases.

### Conclusions From the Analysis

#### Physiological and Mechanical Interpretation of the Results

No significant changes were observed in FVC or FEV1 following the administration of botulinum toxin type A (BTA) to the lateral abdominal muscles. In fact, both parameters showed a slight, statistically non-significant increase. This suggests that the primary inspiratory muscles—such as the diaphragm, intercostals, and accessory muscles—remain unaffected by BTA, and that the rectus abdominis, the principal expiratory muscle, retains its functional integrity. As a result, the basic mechanics of respiration appear preserved and functionally compensated.

A marked increase was noted in PEF (+10.5%) and MMEF (+10.6%), which might be misinterpreted as improved airway function. However, the physiological explanation points elsewhere. BTA-induced relaxation of the lateral abdominal wall (external, internal oblique, and transversus abdominis) reduces intra-abdominal wall tension. This mechanical release allows for greater diaphragmatic descent during inspiration, thereby increasing the initial lung volume (preload) before expiration begins. Consequently, the pressure gradient at the onset of expiration is greater, enabling faster airflow—but not greater exhaled volume.

Thus, the observed increases in PEF and MMEF reflect elevated flow velocity, not improved gas exchange efficiency. They represent enhanced airflow dynamics, not ventilation capacity.

The MEF50 parameter—representing mid-volume expiratory flow—showed a statistically significant increase, supporting this model. It indicates improved flow at intermediate lung volumes, likely driven by the same preload-enhancing mechanism.

In contrast, MEF75 and MEF25 did not show significant changes. MEF75, representing early expiratory flow, remained stable, suggesting the initial phase of expiration is largely unaltered. MEF25, reflecting the terminal phase of expiration, also did not significantly increase—consistent with the hypothesis that the third phase of expiration may be functionally diminished following paralysis of the lateral abdominal muscles. These muscles normally assist in compressing the abdominal contents and elevating the diaphragm in late expiration.

FIVC showed a slight, non-significant decrease (−0.12 L), possibly reflecting a compensatory reduction in inspiratory effort in some patients. However, this finding does not indicate impaired inspiratory function.

In summary, BTA administration does not negatively affect the inspiratory phase of breathing. However, it alters the mechanics of expiration—accelerating the second phase (MMEF) and potentially impairing the third phase (MEF25). As a result, expiratory flow velocity increases (PEF, MMEF, MEF50), but gas exchange efficiency may not improve—and may even decline—if the total exhaled volume is reduced.

Therefore, spirometric “improvements” may in fact mask mechanically driven limitations in breathing that are not detected through standard spirometry. This hypothesis should be the subject of further research, both in the early postoperative period (days 7–10) and in the long term, after the effects of botulinum toxin have fully subsided.

## Discussion

To date, most studies on the preoperative use of botulinum toxin type A (BTA) in patients undergoing complex abdominal wall reconstruction have focused on its effect on lateral muscle relaxation and its role in facilitating fascial closure. The primary endpoints in the literature are typically the extent of rectus muscle medialization and the reduced need for component separation techniques [5, 6, 9]. However, there is a clear gap in the analysis of BTA’s impact on respiratory physiology, particularly in the context of dynamic spirometric parameters.

Although rare reports have suggested possible respiratory compromise following BTA injections [13], no study to our knowledge has provided a systematic, spirometry-based assessment of how muscle paralysis might alter breathing mechanics in the perioperative period. The current study demonstrates that despite seemingly improved airflow metrics (PEF, MMEF), actual air exchange capacity - as reflected by FVC - may decline due to loss of the third phase of expiration. These findings introduce a novel and clinically relevant perspective that has not been addressed in previous literature.

Given the lack of standardized spirometric protocols capable of detecting late expiratory phase deficits, our results highlight the need for refined diagnostic approaches when evaluating respiratory safety and functional outcomes following BTA use in abdominal wall reconstruction.

Two patients in the study had chronic respiratory conditions (one with asthma and one with COPD). Their spirometry results before and after BTA administration did not differ meaningfully from those of the broader cohort. No adverse respiratory outcomes were observed in these cases, but the small sample size precludes subgroup analysis.

## Summary and Generalisability

The findings of this study suggest that the administration of botulinum toxin type A (BTA) is safe from a respiratory perspective, as it does not impair core spirometric parameters such as FVC or FEV1. In fact, both values showed slight, non-significant increases post-administration. Notable changes were observed in flow-related parameters including PEF, MMEF, and MEF50, some of which reached statistical significance. These alterations are best interpreted as mechanical effects—most likely stemming from greater diaphragmatic excursion during inspiration, enabled by reduced tension in the lateral abdominal wall. This mechanical shift likely results in increased lung preload and a steeper pressure gradient during the initial phase of expiration, thereby enhancing airflow velocity. However, these improvements in flow do not appear to correspond with improved gas exchange or total ventilatory output, as total exhaled volume remained unchanged. Moreover, the loss of active contraction in the lateral abdominal muscles may diminish the third phase of expiration, potentially affecting diaphragmatic positioning and long-term respiratory mechanics. These observations underscore the limitations of standard spirometry in capturing subtle or late-phase changes in respiratory dynamics. Future studies employing more advanced tools—such as body plethysmography or diaphragmatic imaging—are needed to better evaluate the true physiological impact of BTA on respiratory function, particularly in the postoperative setting.

## Conclusion

This study demonstrates that BTA, when used preoperatively in patients with large abdominal wall hernias, does not negatively impact core respiratory function as assessed by spirometry. While certain flow-related parameters showed statistically significant increases, these changes appear to reflect mechanical shifts rather than functional improvement or impairment. Spirometry alone may not be sufficient to capture the nuanced effects of BTA on breathing dynamics, particularly during the late expiratory phase. Further research using more advanced diagnostic tools is needed to fully understand the respiratory implications of BTA and to determine its role in guiding patient selection and perioperative care.

## Data Availability

The raw data supporting the conclusions of this article will be made available by the authors, without undue reservation.
